# Inhibitory Effect of Three *C*-glycosylflavonoids from *Cymbopogon citratus* (Lemongrass) on Human Low Density Lipoprotein Oxidation

**DOI:** 10.3390/molecules14103906

**Published:** 2009-09-30

**Authors:** Roxana Orrego, Elba Leiva, José Cheel

**Affiliations:** 1 Department of Clinical Biochemistry and Immunohematology, University of Talca, Talca, Chile; E-Mail: eleivam@utalca.cl; 2 Department of Pharmacognosy, Faculty of Pharmacy, Charles University, Heyrovského 1203, 500 05 Hradec Králové, Czech Republic; E-Mail: cheej7aa@faf.cuni.cz

**Keywords:** flavonoids, lemongrass, oxidized LDL, antioxidant, *Cymbopogon citratus*

## Abstract

This study assessed the inhibitory effect of three *C*-glycosylflavonoids from *Cymbopogon citratus* leaves − isoorientin (**1**), swertiajaponin (**2**) and isoorientin 2"-*O*-rhamnoside (**3**) − on human LDL oxidation. Isolated LDL was incubated with compounds **1**-**3** and the kinetics of lipid peroxidation were assessed by conjugated diene and malondialdehyde-thiobarbituric acid reactive substances (MDA-TBARS) formation after addition of copper ions. Significant differences (p < 0.05) between the lag time phase of the control and the lag time phase in the presence of the compounds **1** (0.25 µM) and **2** (0.50 µM) were observed. After five hours of incubation all three compounds showed a significant inhibitory effect on MDA-TBARS formation with respect to the control. After six hours of incubation only compound **1** kept a remarkable antioxidant effect. This study demonstrates that isoorientin (**1**) is an effective inhibitor of *in vitro* LDL oxidation. As oxidative damage to LDL is a key event in the formation of atherosclerotic lesions, the use of this natural antioxidant may be beneficial to prevent or attenuate atherosclerosis.

## Introduction 

Oxidative damage to biological structures by free radicals has been implicated in a variety of human diseases [1]. Evidence from both *in vitro* and *in vivo* studies suggest that oxidation of low density lipoprotein (LDL), the major cholesterol-carrier in human plasma, contributes critically to human atherosclerosis [2,3], a disorder considered to be the major cause of morbidity and mortality from cardiovascular disease [4]. It is postulated that genesis of atherosclerosis would be intimately connected to the oxidation of plasmatic lipoproteins and the outbreak of several phenomena that involve the participation of different cellular structures and compartments of blood vessels, extracellular matrix and circulating monocytes. In relation to the oxidation of plasmatic lipoproteins leading to changes in their chemical structure, one of the most affected is LDL [5]. During this process, the polyunsaturated fatty acids that make up these particles are oxidized producing lipidic hydroperoxides [6]. According to an earlier report, the progression of these injuries could be slowed using antioxidants such as probucol, vitamin E and butylhydroxytoluene (BHT) [7]. Some investigations suggest that the LDL oxidation starts after the depletion of the endogenous lipophilic antioxidants such as α-tocopherol, β-carotene and ubiquinol [8,9]. Therefore, additional sources of natural antioxidants would be required. 

Flavonoids, which are found abundantly in edible plants, may play a critical role in the prevention of cardiovascular disease by decreasing oxidative damage to LDL [9]. In a recent report from the World Health Organization, the intake of flavonoids was associated with a decreased risk of cardiovascular disease [10]. Among these natural products, *C*-glycosylflavonoids are of particular interest because of their limited occurrence in plants and their potent antioxidant activity [11,12]. Recently, we reported the isolation of five *C*-glycosylflavonoids (orientin, isoorientin, isoscoparin, swertiajaponin and isoorientin 2"-*O*-rhamnoside) from *C. citratus* leaves. This plant is widely employed in the traditional medicine of tropical and subtropical countries and it is also commonly used as an aromatic and pleasant-tasting herbal tea [11]. Its isolated compounds were shown to substantially inhibit the lipoperoxidation in membranes of erythrocytes; however, their ability to protect human LDL from oxidative attack has still not been described. For this reason, the present study was undertaken to ascertain the inhibiting effect of isoorientin, swertiajaponin and isoorientin 2"-*O*-rhamnoside on human LDL oxidation.

## Results and Discussion

**Figure 1:**
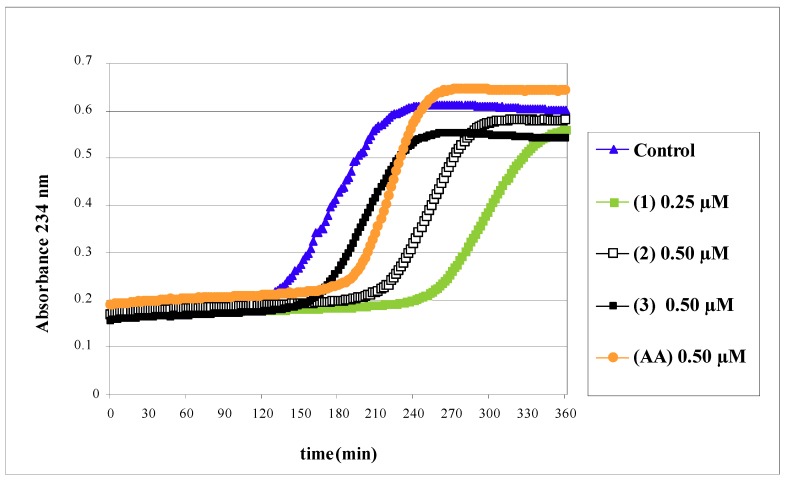
Effects of isoorientin (**1**), swertiajaponin (**2**) and isoorientin 2"-*O*-rhamnoside (**3**) on copper-mediated LDL oxidation by measuring the increase in absorbance at 234 nm due to the conjugated diene formation **AA**: Ascorbic acid. The graphic is representative of four separate experiments.

The formation of conjugated dienes from the LDL lipid fraction provides information about its susceptibility to oxidation, a process that happens permanently in live organisms and is called lipid peroxidation. The prolongation of the lag time phase means an increase of LDL resistance to lipid peroxidation. Isoorientin (**1**), swertiajaponin (**2**) and isoorientin 2"-*O*-rhamnoside (**3**) were evaluated for their capacity to prolong the lag time phase of LDL during oxidation. Significant differences between the lag time phase of a control sample and the lag time phase in the presence of compounds **1** and **2** were found (Figure 1). The inhibitory effect of the flavonoids and ascorbic acid (AA) on the formation of conjugated dienes varied greatly (25-100%, p < 0.05), as observed in Table 1. The highest effect (100%) was exhibited by compound **1** at 0.25 µM, followed by compound **2** (42.7%) at 0.50 µM, whereas compound **3** displayed no significant effect at 0.50 μM. AA showed a weak effect (25%).

**Figure 2:**
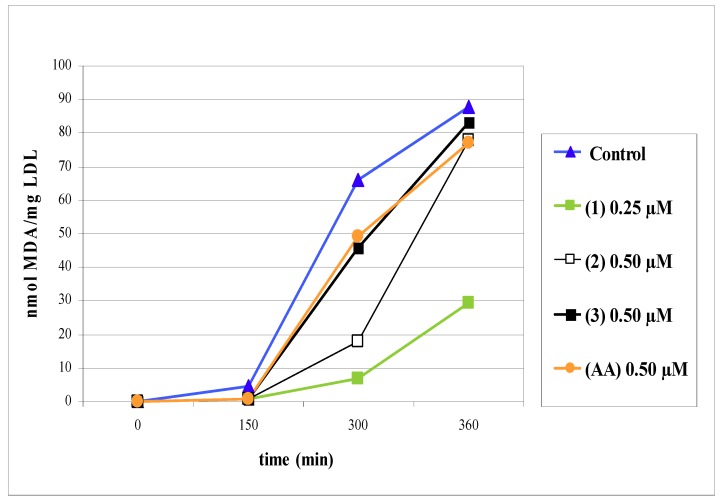
Effects of isoorientin (**1**), swertiajaponin (**2**) and isoorientin 2"-*O*-rhamnoside (**3**) on MDA formation (nmol of MDA-TBARS/LDL protein) during copper-induced LDL oxidation. **AA**: Ascorbic acid. The graphic is representative of six separate experiments.

The LDL oxidation was also determined by measuring the amount of MDA as thiobarbituric acid reactive substances (TBARS). After five hours of incubation with flavonoids, there was a significant decrease in the MDA-TBARS formation (Figure 2). Compound **1** showed the highest inhibiting effect (89%) on MDA-TBARS formation, followed by compounds **2** (73%) and **3** (28%). Ascorbic acid (AA) showed a weak inhibiting effect (25%). After six hours of incubation only compound **1** retained a significant inhibiting effect (65.7%), as observed in Table 1.

Although many elements are involved in the atherosclerosis process, the oxidative modification hypothesis has been the central focus of innumerable studies. This theory claims that the oxidative modification of LDL and other lipoproteins is a central and almost obligatory element in the development of atherosclerosis. This theory also implies that other molecules, the antioxidants, are can avoid the occurrence of oxidative modifications of LDL and therefore are effective in the suppression of atherosclerosis [13,14]. In the present investigation, the antioxidant effects of compounds **1**, **2** and **3** against copper ions-induced LDL oxidation are shown for the first time. It is known that many flavonoids can chelate copper or ferrous ions and part of their antioxidative effect on LDL may be attributed to this chelating property [15]. Other flavonoids have exhibited their inhibiting effect on LDL oxidation by protecting LDL associated carotenoids [8].

**Figure 3:**
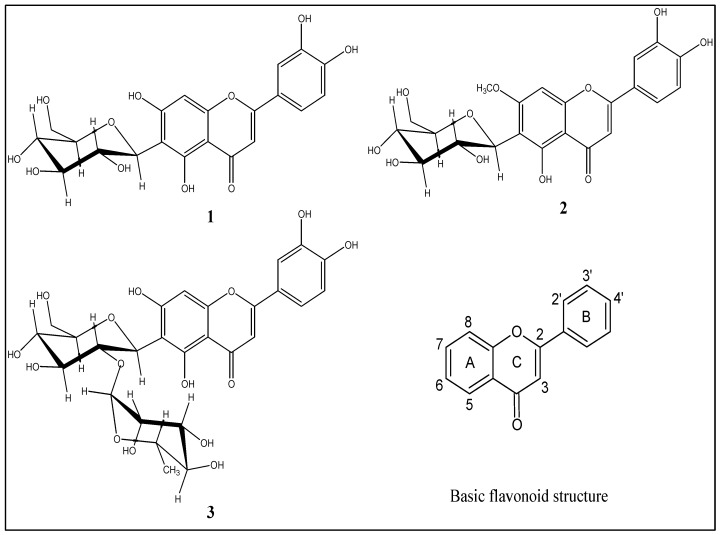
Chemical structures of isoorientin (**1**), swertiajaponin (**2**) and isoorientin 2"-*O*-rhamnoside (**3**).

The present study reveals that the antioxidant activities of compounds **1**, **2** and **3** on LDL oxidation appear to reside predominantly in the hydroxyl groups at position 7 on the A ring and at positions 3′, 4′ on the B ring (Figure 3), which is consistent with a previous report [9]. Compound **1**, the best antioxidant, has the sugar attached at C-6, while methoxylation at C-7 in compound **2** or an additional sugar (rhamnose) at C-6 in compound **3** reduced the antioxidant effect. Compounds **1** and **3** have an equal number of OH groups but the difference is in their glycoside substituent. The monoglycosylated flavonoids **1** and **2** were shown to be more active against LDL oxidation than the diglycosylated flavonoid **3**. In a prior study [12], a glycosylated derivative of isoorientin inhibited TBARS formation during copper-mediated LDL oxidation with IC_50_ value of 5.6 μM. In the current study, isoorientin inhibited the TBARS formation with IC_50_ value below 0.25 μM (Table 1). It may be hypothesized that compounds with less hydrophilic character are more available to the lipid structure of LDL and thus protecting the lipids from oxidation.

It is noteworthy that compounds **1** and **2** displayed a remarkable antioxidant effect at smaller concentrations than those reported by other authors [16]. Most studies indicate that plasma flavonoid concentrations of about 1 μM are obtained following normal intake of foods rich in phenolics. [17]. In the present study a considerable antioxidant effect of isoorientin (**1**) was observed, even at 0.25 μM. This fact is very favorable since exposure to increased levels of flavonoids has been associated to toxic effects [18]. There is little information in the literature on the bioavailability and metabolism of *C*-glycosylflavonoids. Most pharmacokinetic studies have been mainly focused on *O*-glycosylflavonoids. In a recent pharmacokinetic study [19], orientin (an isomer of isoorientin) was quickly distributed and eliminated within 90 min after intravenous administration. The tissue distribution results showed that liver, lung and kidney were the major distribution tissues of orientin in rats, and that orientin had difficulty in crossing the blood-brain barrier. It was also found that there was no long-term accumulation of orientin in rat tissues.

In summary, the present study showed favorable effects of isoorientin on LDL oxidative susceptibility. This flavonoid could be a potential candidate to be used as an antiatherogenic agent. Further studies are needed to ascertain the *in vivo* antioxidant effect and to determine the action mechanism of this promising compound. The content of isoorientin should be considered when evaluating the antiatherogenic effect of *C. citratus* and its nutraceutical formulations.

## Experimental

### Flavonoids from C. citratus

The *C*-glycosylflavonoids isoorientin (**1**), swertiajaponin (**2**) and isoorientin 2"-*O*-rhamnoside (**3**) were previously isolated from *C. citratus* (lemongrass) leaves and their structures were determined using NMR spectroscopy [11]. 

### LDL isolation from human plasma

Peripheral venous blood was collected in tubes containing EDTA (1 mg/mL) and plasma was isolated by centrifugation at 550 X g for 15 min at 20 ºC. The LDL protein was isolated from plasma by differential ultracentrifugation, according to the method of Galle and Wanner [20] and the protein concentration was determined using the Lowry assay [21].

### Formation of conjugated dienes

The degree of LDL oxidation was measured with respect to formation of conjugated dienes by monitoring the change in absorbance at two min intervals at 234 nm using a Genesys 10 UV spectrophotometer [22]. The LDL (30 µg/mL) were incubated with 10 μM cooper sulfate in absence (control) or presence of test compounds in NaCl 0.15 M, for 6 h at 25 ºC. The lag time in the presence or absence of the test compounds was determined to be the intercept of the slopes for the lag and propagation phases and was compared with the control oxidized LDL to determine the percentage of LDL oxidation inhibition. Data were expressed as median ± IR (n = 4). Ascorbic acid (AA) was used as a reference antioxidant.

### Malondialdehyde content in LDL fraction (MDA-TBARS)

To evaluate the extent of LDL oxidation, the malondialdehyde (MDA) formed by copper-mediated LDL oxidation was measured as thiobarbituric acid reactive substances (TBARS) [23]. The LDL (80 µg) was incubated for six hours at 25ºC with CuSO_4_ (10 µM) and test flavonoids. Following oxidation, 500 µL samples were taken at different times and then incubated for 30 minutes at 90 °C with 1 mL reactive thiobarbituric acid 0.67 g/dL and 1 mL of trichloroacetic acid 50 g/dL. The MDA-TBARS content was measured at 532 nm. Results were expressed as nmol of MDA-TBARS/milligram of LDL protein and as percent inhibition on MDA-TBARS formation relative to control. Data were expressed as mean ± SD (n = 6). Ascorbic acid (AA) was used as a reference antioxidant.

### Statistical analysis 

To determine whether there was any difference between values of TBARS, a one-way analysis of variance (ANOVA MR) was applied. For conjugated dienes Kruskal-Wallis was applied. The differences between means were determined using Tukey tests for multiple comparisons. Value of (p < 0.05) was considered to be significant. The Statistical Package SPSS version 15.0 was used to analyze the data.

## Figures and Tables

**Table 1:** Inhibitory effect (%) of isoorientin (**1**), swertiajaponin (**2**) and isoorientin 2"-*O*-rhamnoside (**3**) on TBARS and conjugated diene formation during copper-mediated LDL oxidation.

Sample	% Inhibition TBARS (5 h)	% Inhibition TBARS (6h)	% Inhibition Conjugated dienes (6h)
Control	0.0	0.0	0.0
(1) 0.25 μM	88.9^a^ ± 5.7	65.7^a^ ± 8.9	99.6^a^ ± 19.3
(2) 0.5 μM	72.6^a^ ± 2.9	10.8 ± 8.5	43.2 ^a^ ± 34.1
(3) 0.5 μM	28.1^a^ ± 20.0	8.2 ± 5.9	-3.6 ± 15.0
(AA) 0.5 μM	25.1^a^ ± 18.3	12.0 ± 10.6	25.0 ± 28.0

Data on inhibition of TBARS and conjugated dienes formation are expressed as mean ± SD (n = 6) and median ± IR (n = 4), respectively. AA: Ascorbic acid. Superscript (a) indicates significant differences compared to control at (p < 0.05).
